# ESD with elastic ring traction is more effective and safer than conventional ESD in large proximal colon neoplastic lesions: a retrospective cohort study (with video)

**DOI:** 10.1007/s00464-023-10445-8

**Published:** 2023-10-31

**Authors:** Sikong Yinhe, Jiao Yang, Zhang Aijun, Li Ruyuan

**Affiliations:** 1 Department of Gastroenterology, Qilu Hospital (Qingdao), Cheeloo College of Medicine, Shangdong University, 758 Hefei Road, Qingdao, Shangdong China; 2 Department of General Surgery, Qilu Hospital (Qingdao), Cheeloo College of Medicine, Shangdong University, 758 Hefei Road, Qingdao, Shangdong China

**Keywords:** Proximal colon, ESD, Elastic ring, Traction

## Abstract

**Background and aims:**

Colorectal endoscopic submucosal resection (ESD), especially ESD in proximal colon, has always been challenging. We invented a novel elastic ring as a traction method to facilitate ESD. Our study aims to compare the safety and effectiveness of ESD with in vivo traction and conventional ESD without traction in the treatment of large proximal colon neoplastic lesions.

**Methods:**

This retrospective cohort study included consecutive patients with large (≥ 20 mm in their maximal diameter) proximal colon neoplastic lesions who underwent ESD with in vivo elastic ring traction or conventional ESD without traction in our endoscopy center between June 2018 and April 2022 by one experienced endoscopist.

**Results:**

The ESD with traction group has lower overall complication rate and lower perforation rate than those in the conventional ESD group (0% vs 14.71%, *P* = 0.021; 0% vs 11.76%, *P* = 0.048, respectively), and the differences are statistically significant. Although there are no significant differences in the rates of en bloc resection and R0 resection and bleeding rate, ESD with traction group still had higher en bloc resection and R0 resection rates and lower bleeding rate than conventional ESD group without traction (100% vs 94.12%, *P* = 0.226; 94.59% vs 85.29%, *P* = 0.189, 8.82% vs 2.70%, *P* = 0.276, respectively).

**Conclusion:**

ESD with elastic ring traction is potentially more effective and safer than conventional ESD in the treatment of large proximal colon neoplastic lesions. Further large, prospective controlled studies are needed to fully evaluate this novel method.

**Supplementary Information:**

The online version contains supplementary material available at 10.1007/s00464-023-10445-8.

Endoscopic submucosal resection (ESD) is widely used in many specialized centers to treat colonic neoplastic lesions due to its low morbidity than conventional surgery [1]. After its first introduction in 1988, ESD has evolved significantly with development of new tools as well as techniques [2]. ESD has an important role in en bloc removal of advanced gastrointestinal lesions. The primary advantage of ESD is high R0 resection rate and en bloc resection rate, especially in large lesions [3, 4]. However, ESD requires high technical skills and has long operation time and relatively high complication rates, such as perforation and delayed bleeding. As reported, ESD of colon, especially of proximal colon, is more challenging than ESD in other gastrointestinal sites, due to the thinner colonic walls, the presence of flexures and folds, and peristaltic movements [5, 6].

The key of safe ESD is to adequately expose the submucosal layer and the cutting line for precise dissection during the whole procedure [7]. To overcome the challenges we have encountered, several novel traction devices have been introduced and developed to facilitate ESD, both in vivo and ex vivo. The existing traction methods include clip line traction, gravity-based traction, S-0 clip, and double-clip traction, which have been proven effective and safe in colorectal ESD [8–12]. However, clip line traction is troublesome during colorectal ESD, since withdrawal of the endoscope is required to attach a string to an endoclip outside the patient [8]. Besides, the main disadvantage of magnetic bead-assisted ESD was that it could only be utilized for distal colonic lesions, as there would not be enough working space in the proximal colon [10]. As before mentioned, S-O clip and double-clip traction have been proven effective in colorectal ESD [11, 12]; however, the data of its effectiveness in proximal colonic ESD are very limited.

Recently, we reported a case series which proved ESD with elastic ring traction is effective and safe in proximal colon neoplastic lesions [13]. There are several studies which involve ESD in proximal colon; however, they are mainly ex vivo study or feasibility study [14, 15]. The controlled studies concerning proximal colonic ESD with traction in vivo are still lacking. Therefore, this retrospective cohort study was designed to compare the safety and effectiveness of ESD with in vivo traction and ESD without traction in the treatment of proximal colon neoplastic lesions.

## Materials and methods

### Patients

This retrospective study included patients with lesions in the proximal colon who underwent ESD at the Department of Gastroenterology, Qilu Hospital of Shandong University, Qingdao, China, from June 2018 to April 2022. ESD surgery of all patients was performed by the same doctor, an associate chief physician who has 6 years of experience in ESD performance. Informed consent was obtained from all included patients.

The inclusion criteria were as follows: (1) patients who were over 18 years of age; (2) patients who had large sessile colorectal polyps or laterally spreading tumors in the proximal colon; and (3) the lesions were ≥ 2.0 cm in diameter. The exclusion criteria were as follows: (1) patients with abnormal coagulation function, incomplete clinical data; (2) patients who underwent hybrid ESD; (3) patients with failed ESD (underwent surgery because of severe perforation during ESD); (4) patients with the pathology of deeply submucosal invasive carcinomas; and (5) patients with the pathology of hyperplastic polyps or inflammatory polyps.

The patients were divided into two groups based on using elastic ring traction or not: traction group and conventional group without traction.

### Outcome measurements

Demographic data (age, sex) and lesion information (location, size, pathological diagnosis) were collected. Primary outcome of this study was the rate of complications, including perforation and bleeding. Immediate perforation was defined as perforation during ESD, and the definition of delayed perforation was perforation after the procedure, and free air could be detected by radiography. Immediate bleeding was defined as bleeding occurred during the procedure that needed to be controlled using hemostatic forceps. Delayed bleeding was defined as bleeding symptoms within 30 days after ESD.

The secondary outcomes were the rates of en bloc resection, R0 resection (tumor-free vertical and lateral margins), and tumor recurrence. Tumor recurrence was defined if biopsy samples of follow-up endoscopy revealed the presence of tumor cells. Other outcomes include dissection time and dissection speed. Dissection time was measured from submucosal injection to detachment of the tumor, which included the extra time for applying elastic ring and endoclip. Dissection speed was defined as area of resected specimen/dissection time (mm^2^/min). The area of tumor was measured by half of length times half of width multiplied by 3.14(*π*). The study protocol was approved by the Biomedical Research Ethics Committee of the Qilu Hospital, Cheeloo College of Medicine, Shangdong University.

### Endoscopic procedures

The ESD procedure was performed under general anesthesia with intubation, and colon inflation was done using CO2. We used therapeutic colonoscopes (Pentax i-5000 main machine, Pentax EG29-i10 endoscope) with a 4-mm distal cap attached (D-201-11304, Olympus). For all procedures, we used Dual knife (Olympus), rotatable soft tissue clip with repetitive open-close function (Nanjing Minimally Invasive Company, China), and a VIO-200S high-frequency electric knife (ERBE Company, Germany) and a Micro-Tech, an elastic ring (patent number: ZL202020,016,729.9). Hydroxyethylamidon mixed with indigo carmine was used for initial submucosal injection.

**Fig. 1:**
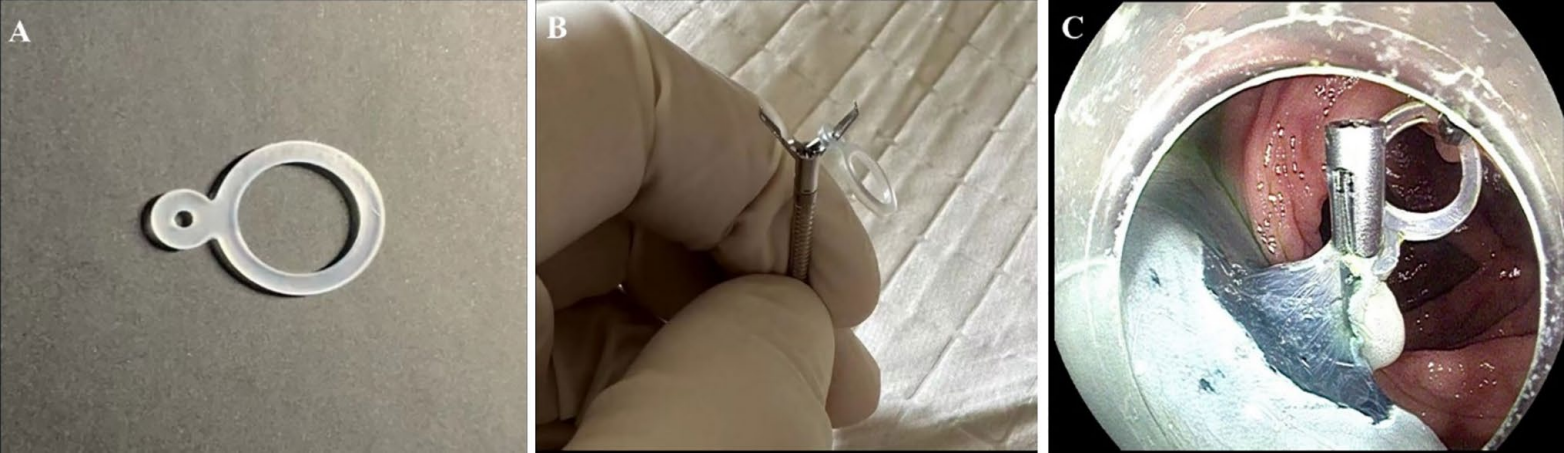
**A** The elastic ring we invented was constructed by two circles combined, and the diameters of the two circles are 2 mm and 8 mm, respectively. It is deformable and can pass through the biopsy channel. **B** We use the endoclip device to clamp the small circle of the elastic ring. **C** Clear endoscopic vision of how the elastic ring system works. The elastic ring and endoclip system were fixed on the anal side of the lesion, another endoclip was applied to clamp and tighten the free end of the elastic ring, and then the endoclip was fixed on the opposite side of the colonic wall

**Fig. 2:**
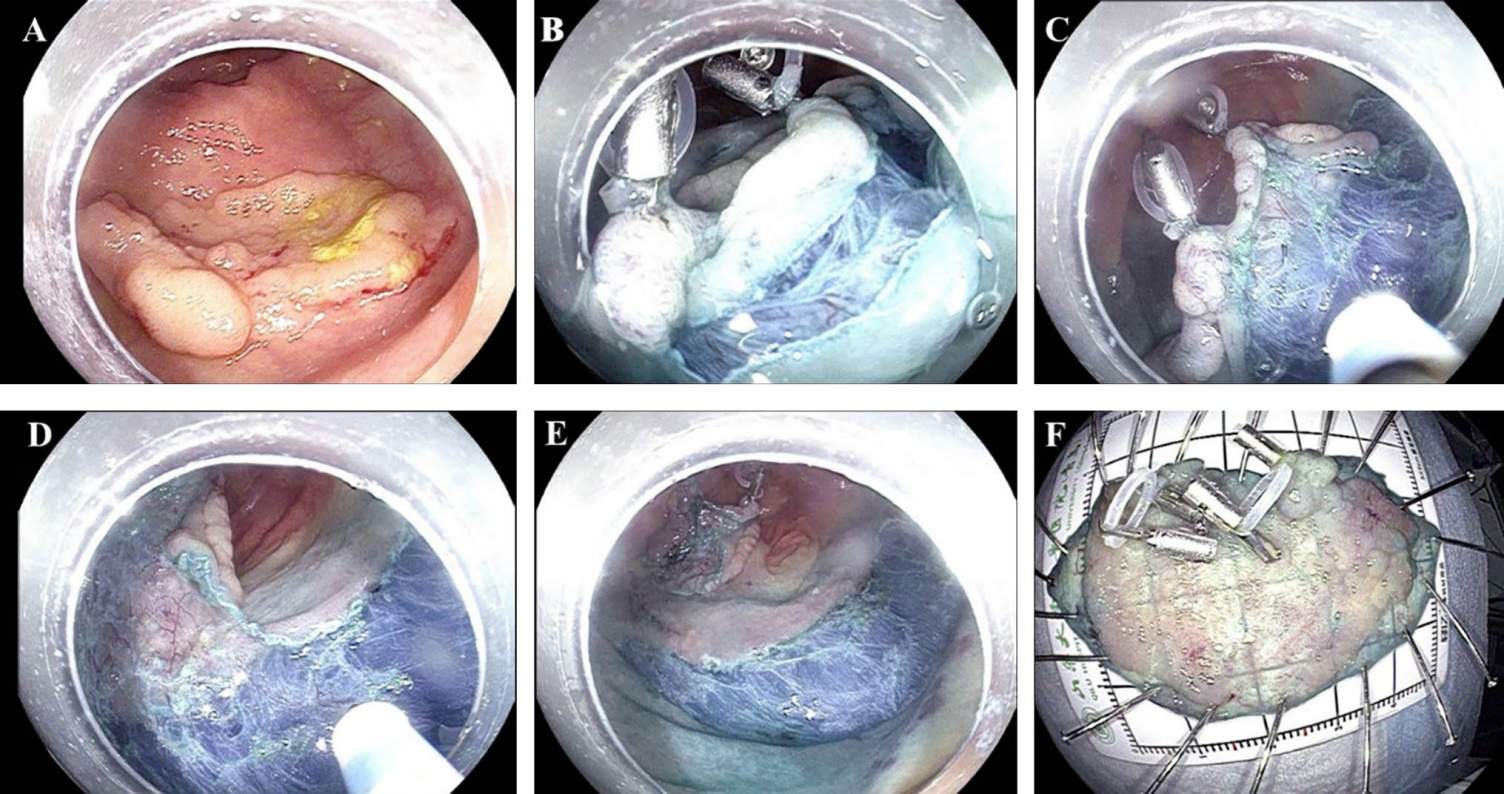
Effective exposure of the submucosal layer achieved by elastic ring traction in a large laterally spreading tumor. **A** The large granular lateral spreading tumor in ascending colon (size 4.5 × 3.0 cm). **B** Unclear visualization of the submucosal layer noted before traction. **C** Adequate submucosal exposure of the local region achieved by applying two elastic ring system. **D** Clear visualization of the vessels. **E** The mucosal defect after en bloc resection. **F** The resected lesion

**Fig. 3:**
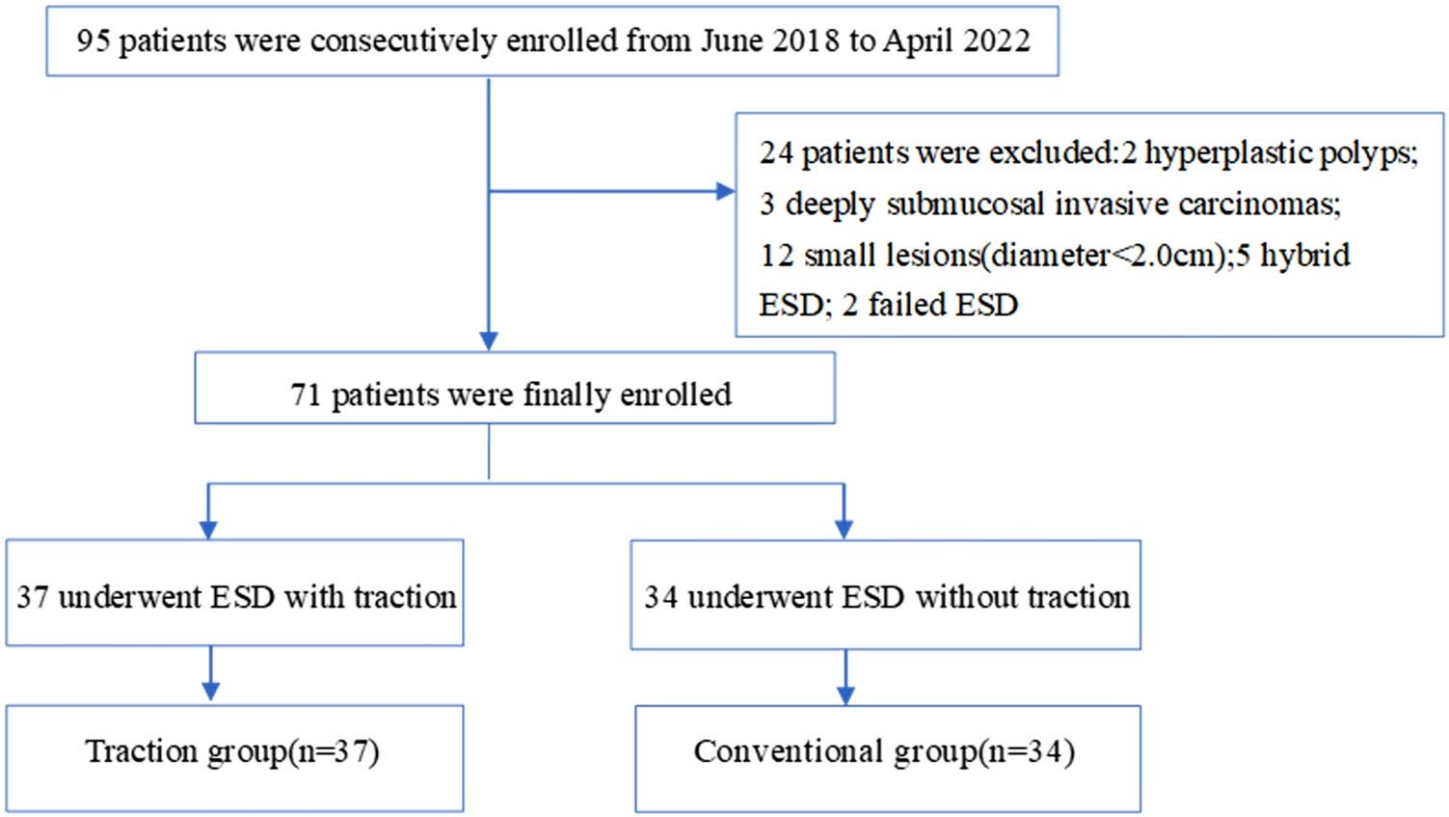
Flow diagram of patient selection into traction group and non-traction group

The following devices were used for the ESD procedure: The lesion boundary was identified after staining the lesion and subsequently magnifying the endoscopic images. Epinephrine and methylene blue saline (1:10,000) were injected submucosally at the root of the lesion to lift the lesion fully. The mucosa around the lesion was resected. An endoclip was used to clamp the elastic ring, sent into the intestinal cavity via the biopsy channel, and fixed on the anal side of the lesion. Another endoclip was applied to clamp and tighten the free end of the elastic ring. It was fixed on the mucosa on the opposite side of the lesion, and the lesion was pulled up to fully expose the submucosa and cutting line. The lesion was then completely resected. Then, electrothermal hemostatic forceps were used to manage vascular stump bleeding. An endoloop or hot hemostatic forceps was used to assist in releasing from the elastic ring to completely resect the lesion (Figs. 1, 2, 3).

### Statistical analysis

Data were statistically analyzed using SPSS 25.0 (IBM, Armonk, NY, USA). Continuous variables were tested for normality. Normally distributed variables were expressed as means ± standard deviation, and Student *t* test or Whitney *U*-test were used to compare the two groups. Data with non-normal distribution were expressed as medians (range). The classification variables were expressed as *n* (%), and the two groups were compared by Pearson Chi-square test or Fisher exact test. *P* < 0.05 was considered statistically significant.

## Results

### Demographic characteristics

A total of 95 patients from June 2018 to April 2022 of our center performed by one experienced physician were consecutively enrolled in our study, then we excluded 2 patients due to the histopathology of hyperplastic polyps, and 3 patients due to the histopathology of deeply submucosal invasive carcinomas, 11 patients due to tumor size less than 2.0 cm, 5 patients who underwent hybrid ESD; and 2 patients with failed ESD. Finally, a total of 71 patients were included in our study. There were 37 patients underwent proximal colonic ESD with traction group and 34 patients underwent proximal colonic ESD without traction group, in which the differences in patient age, sex, tumor location, and size (including length, width, area) were well balanced (shown in Table 1).

**Table 1:** Baseline characteristics of the patients enrolled and colorectal lesions of traction group and conventional group without traction

	Traction group (*n* = 37)	Conventional group (n = 34)	*P* value
Age	60.92 ± 9.40 (41–76)	61.56 ± 11.48 (30–82)	0.640^a^
Sex	Male 27	Male 25	0.958^c^
	Female 10	Female 9	
Location	Ascending 13	Ascending 20	0.095^c^
	Liver flexure 4	Liver flexure 4	
	Transverse 20	Transverse 10	
Length (mm)	24.46 ± 6.72 (20–52)	24.89 ± 6.40 (20–45)	0.661^d^
Width (mm)	19.78 ± 6.30 (10–35)	21.12 ± 4.44 (15–31)	0.162^d^
Area (mm^2^)	402.46 ± 255.10 (188.40–1306.24)	431.08 ± 210.17 (235.50–1059.75)	0.152^d^

Age, length, width, and area are expressed as mean ± standard deviation (minimum, maximum); area is measured by half of the maximum diameter times half of the minimum diameter multiplied by 3.14

^a^Student *t* test

^c^Pearson Chi-square

^d^Whitney *U*-test

The details of ESD procedures in the two groups are shown in Table 2. All the procedures were accomplished by one experienced endoscopist and using Dual knife. Although without statistical differences, the dissection time and dissection speed were improved when using elastic ring traction (34.68 min vs. 43.12 min, *P* = 0.128; and 13.07 mm^2^/min vs. 11.89 mm^2^/min, *P* = 0.629, respectively).

**Table 2:** Procedure details in the proximal colonic ESD with elastic ring traction and conventional ESD without traction

	Traction group (*n* = 37)	Conventional group (*n* = 34)	*P* value
Endoscopist Dr. Li	37 (100%)	34 (100%)	1.000^a^
Endoscopic knife Dual knife	37 (100%)	34 (100%)	1.000^a^
Use of hemostatic forceps	1 (2.70%)	5 (14.71%)	0.081^a^
Dissection time (min)	34.68 ± 19.38 (11–114)	43.12 ± 25.40 (16–120)	0.128^b^
Dissection speed (mm^2^/min)	13.07 ± 6.80 (3.93–28.55)	11.89 ± 6.47 (2.36–38.53)	0.629^b^
Tumor recurrence	0	0	–

Dissection time and dissection speed are expressed as mean ± standard deviation (minimum, maximum); dissection speed is measured by area/dissection time

^a^Fisher’s exact test

^b^Whitney *U*-test

Comparison results of clinical outcomes in the two groups are summarized in Table 2. Although there are no significant differences in the rates of en bloc resection and R0 resection, ESD with traction group still had higher en bloc resection and R0 resection rates than conventional ESD group without traction (100% vs 94.12%, *P* = 0.226; 94.59% vs 85.29%, *P* = 0.189, respectively). Most importantly, the traction group has lower overall complication rate and lower perforation rate than those in the conventional group (2.70% vs 20.59%, *P* = 0.020; 0% vs 11.76%, *P* = 0.048, respectively), and the differences are statistically significant. The most common complication in the conventional ESD group was perforation (3 immediate and 1 delayed, respectively). All the three patients with immediate perforation recovered from conventional treatment with fasting and antibiotics; however, additional surgery was required for the patient with delayed perforation. The second most common complication was immediate bleeding, immediate bleeding occurred in 3 patients, and hemostatic forceps were needed to coagulate the vessels. There was only one immediate bleeding in the traction group, although the difference is statistically insignificant (2.70% vs 8.82%, *P* = 0.276), we may presume that elastic ring traction could reduce immediate bleeding rate. There was no delayed bleeding in two groups. All patients in the traction group and the conventional group showed no evidence of tumor recurrence at follow-up examination (Table 3).

**Table 3:** Clinical outcomes in the traction group and conventional group

	Traction group (*n* = 37)	Conventional group (*n* = 34)	*P* value
En bloc resection	37 (100%)	32 (94.12%)	0.226^a^
R0 resection	35 (94.59%)	29 (85.29%)	0.181^a^
Complication	1 (2.70%)	7 (20.59%)	0.020^a^
Perforation	0	4 (11.76%)	0.048^a^
Immediate perforation	0	3 (8.82%)	0.102^a^
Late perforation	0	1 (2.94%)	0.479^a^
Bleeding	1	3 (8.82%)	0.276^a^
Immediate bleeding	1 (2.70%)	3 (8.82%)	0.276^a^
Delayed bleeding	0	0	–

^a^Fisher’s exact test

## Discussion

In the present study, the results showed that despite being statistically insignificant, the dissection time and dissection speed were improved when using elastic ring traction, and ESD with traction group had higher en bloc resection and R0 resection rates than conventional ESD group without traction. Of note, the traction group has lower overall complication rate and lower perforation rate than those in the conventional group, and the differences are statistically significant, which provides more evidence that in vivo traction in resecting proximal colonic tumors is effective and safe [13, 14]. Besides, the immediate bleeding rate was lower in the traction group than conventional ESD group, despite of the statistical insignificance. Altogether our results suggested that ESD with elastic ring traction is more effective and safer than conventional ESD in the treatment of large proximal colon neoplastic lesions.

ESD with in vivo elastic ring traction has following advantages. First, elastic ring provides additional traction force to better expose submucosal layer and cutting line, which makes incision and coagulation more precise, and thus, reduce the risk of bleeding and perforation [16, 17]. Second, a major advantage of the elastic ring is its convenience and practicability; unlike the thread-and-clip method [18], there is no requirement of retrieval and reintroduction of the endoscope since the elastic ring is deformable and can pass through the biopsy channel together with endoclip device. And there is no need of patient position changing for adjusting the direction of traction, unlike other gravity-based traction system which is applied widely [19–21]. Besides, the traction force achieved by elastic ring is very gentle; thus, no tearing of the tissue happened in all 37 patients who underwent ESD with elastic ring traction. Of note, when the submucosal space is insufficient during dissection, we can always add another elastic ring to render traction in different directions through the biopsy channel. Third, it is simple to insert and apply the elastic ring and endoclip, having no need for special training. Finally, the cost of this traction system is very low, since it only involves the elastic ring invented by ourselves and endoclip, no other heavy and expensive devices were concerned [22].

Since ESD in the proximal colon requires higher levels of endoscopic skill and experience [23, 24], there are few studies concerning ESD with in vivo traction in proximal colon. To our knowledge, we are the first to demonstrate that ESD with in vivo traction in proximal colon is more effective and safer than conventional ESD in a retrospective cohort study. Besides, the elastic ring is our patent, according to our positive results, this elastic ring is safe and effective, which could be used as a novel traction device in daily clinical course in the future.

There are some limitations in our study. First, our study is a retrospective single center study; thus, the selection bias is inevitable. However, the patients were enrolled consecutively to lower the selection bias, and all the ESD procedures were performed by one experienced endoscopist, which could avoid the bias caused by different performers. Besides, the baseline characteristics of patients and proximal colon neoplastic lesions were well balanced. Second, all the procedures were performed by one endoscopist, although this helps reduce the bias, but it also prevents us from evaluating the generalizability of this particular method in less experienced hands. Further researches should be designed to examine the generalizability and the learning curve of this novel traction device. Finally, the sample size is relatively small. Further large, prospective, controlled, and multicenter studies are warranted to assess the safety and effectiveness of elastic ring traction for large proximal colonic neoplastic lesions.

In conclusion, proximal colonic ESD with in vivo elastic ring traction is potentially more effective and safer than conventional ESD in the treatment of large proximal colon neoplastic lesions. Further prospective multicenter trials are warranted to evaluate the generalizability and applicability of this novel method.

### Supplementary Information

Below is the link to the electronic supplementary material.

Supplementary file1 (MP4 95,201 kb)
